# Investigation of the Physical, Chemical and Microbiological Stability of Losartan Potassium 5 mg/mL Extemporaneous Oral Liquid Suspension

**DOI:** 10.3390/molecules26020301

**Published:** 2021-01-08

**Authors:** Lisa Foley, Jennifer Toney, James W. Barlow, Maura O’Connor, Deirdre Fitzgerald-Hughes, Zebunnissa Ramtoola

**Affiliations:** 1 School of Pharmacy and Biomolecular Sciences, RCSI University of Medicine and Health Sciences, 111 St. Stephen’s Green, Dublin 2, Ireland; lisafoley@rcsi.ie (L.F.); jennifertoney@alumnircsi.com (J.T.); 2 Department of Chemistry, RCSI University of Medicine and Health Sciences, 123 St. Stephen’s Green, Dublin 2, Ireland; jambarlow@rcsi.com; 3 Pharmacy Department, Children’s Health Ireland (CHI) at Crumlin, Dublin 12, Ireland; maura.oconnor@olchc.ie; 4 Department of Clinical Microbiology, Education and Research Centre, Beaumont Hospital, RCSI University of Medicine and Health Sciences, Dublin 9, Ireland; dfitzgeraldhughes@rcsi.com

**Keywords:** losartan, extemporaneous compounding, oral suspension, stability, solubility

## Abstract

Extemporaneous oral liquid preparations are commonly used when there is no commercially available dosage form for adjustable dosing. In most cases, there is a lack of stability data to allow for an accurately assigned shelf life and storage conditions to give greater confidence of product safety and efficacy over its shelf life. The aim of this study was to evaluate the physical, chemical and microbiological stability of an extemporaneous oral liquid suspension of losartan potassium, 5 mg/mL, used to treat paediatric hypertension in Our Lady’s Children’s Hospital Crumlin, Ireland. The losartan content of extemporaneous oral suspensions, prepared with and without addition of water, was measured by UV and confirmed by HPLC analysis. Suspensions were stored at 4 °C and room temperature (RT) and were monitored for changes in; pH, colour, odour, re-dispersibility, Total Aerobic Microbial Count, Total Yeast and Mould Count and absence of *E. coli*. Results showed that suspensions prepared by both methods, stored at 4 °C and RT, were physically and microbiologically stable over 28 days. Initial losartan content of all suspensions was lower than expected at 80–81% and did not change significantly over the 28 days. HPLC and NMR did not detect degradation of losartan in the samples. Suspensions prepared in water showed 100% losartan content. The reduced initial losartan content was confirmed by HPLC and was related to the acidic pH of the suspension vehicle. Physiochemical properties of the drug are important factors for consideration in the selection of suspension vehicle for extemporaneous compounding of oral suspensions as they can influence the quality, homogeneity and efficacy of these preparations.

## 1. Introduction

**Figure 1:**
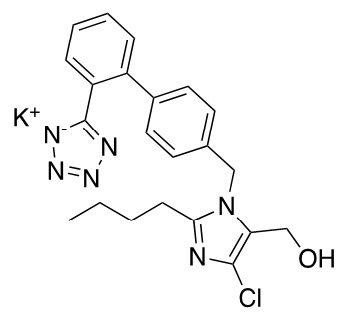
Losartan potassium.

Extemporaneous oral liquid preparations are formulated by pharmacists when there is no commercially available dosage form for adjustable dosing or appropriate dosage form for pharmaceutical care of paediatric patients, elderly patients and patients with specific conditions [1,2]. A major issue arising from this practice is the limited information available to support the stability, storage and shelf life of such products, which are generally assigned an arbitrary shelf-life [3]. In addition, the physical, chemical, and microbiological properties must be considered when formulating extemporaneous preparations, as these can impact on dose uniformity, stability, and storage conditions [4]. Losartan potassium (C_22_H_22_ClKN_6_O) is a specific non-peptide angiotensin II receptor blocker (ARB) used in the treatment of hypertension (Figure 1).

The drug is orally active and is available in tablet form in doses ranging from 12.5 to 100 mg for administration in adults. Losartan is reported to have demonstrated benefit in children aged 1 month to 16 years [5]. Losartan was the first ARB approved for paediatric hypertension by the U.S. Food & Drug Administration (FDA) in 2004, in response to regulatory initiatives for paediatric clinical trials, by the FDA and the European Medicines Agency (EMA) [6]. Following completion of the required paediatric clinical trials as part of the commitment of Merck Sharp & Dohme Ltd. (MSD), to its paediatric implementation plan (PIP) for Cozaar^®^, losartan was recommended in children aged 6-18 years, at a dosage of 0.7 mg/kg once daily (patients >20 to <50 kg; max 25 mg) and for patients >50 kg, the recommended dose was 50 mg once daily [6,7]. The dosage should be adjusted according to response [6,7]. In 2009, following EMA approval, Cozaar^®^ 2.5 mg/mL sachet for oral suspension (Cozaar^®^ 2.5 mg/mL suspension) was licensed in a number of EU countries [8]; however, the product was discontinued in Ireland in 2019 [9]. Due to the limited availability and/or discontinuation of Cozaar^®^ 2.5 mg/mL suspension, extemporaneous compounding of losartan oral suspension is carried out in various hospitals, both in Ireland and globally. Extemporaneous oral suspensions of 2.5 mg/mL and 5 mg/mL losartan potassium are compounded for individualised dosing of specific patients. A shelf–life of 28 days is assigned to the 2.5 mg/mL suspension, stored refrigerated, as per published monographs [10,11], and an arbitrary shelf life of 7 days is assigned to the 5 mg/mL preparation stored refrigerated, in the absence of stability data. Losartan potassium has a pKa of 4.9 and a pH dependent solubility [12]. Losartan has been shown to degrade under acidic conditions, under thermal stressing at 70 °C, by oxidation and is also photodegradable [13,14].

The objective of this study was to investigate the physical, chemical and microbiological stability of the losartan potassium 5 mg/mL extemporaneous suspensions over 28 days under refrigerated conditions (4 °C) and at room temperature (RT), in order to assign an expiry date and suitable storage conditions for the extemporaneous suspension. Losartan potassium (Losartan) extemporaneous oral suspensions were prepared by the method used in Children’s Health Ireland at Crumlin, Ireland (method A) and as per published monographs [10,11] (method B), without and with addition of water, respectively. Losartan (as losartan potassium) concentration was measured over 28 days, using UV spectroscopy and a stability indicating HPLC method [15,16]. Suspensions were monitored for changes in pH, colour, odour and re-dispersibility. The Total Aerobic Microbial Count (TAMC), Total Yeast and Mould Count (TYMC) and absence of *E. coli* of the suspensions were measured over the 28 days, to assess microbiological stability according to the criteria of the European Pharmacopoeia (EP).

## 2. Results

### 2.1. pH, Visual Appearance and Organoleptic Properties of Suspensions

**Figure 2:**
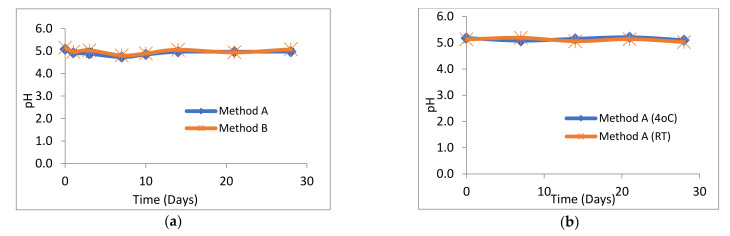
pH of losartan suspensions stored over 28 days. (*n* = 3, *±*SD, *p* > 0.05) (**a**) prepared by methods A and B and stored at 4 °C, (**b**) prepared by method A and stored at 4 °C or room temperature (RT).

The pH of all losartan suspensions was in the range 4.73–5.21 over the 28-day study period. The pH profiles for suspensions prepared by method A and method B were almost identical (Figure 2a). Similarly, there was no significant difference in the pH profiles of losartan suspensions prepared by method A and stored at either 4 °C or at RT (Figure 2b). No change in the pale pink/white colour or sweet cherry odour of losartan suspensions or suspending vehicle was observed throughout the 28 days at both 4 °C and RT. Losartan suspensions stored unopened at 4 °C had a pH of 5.15 ± 0.15. All suspensions were easily re-dispersed and no visible degradation was observed over the 28 days.

### 2.2. Chemical Stability of Losartan Suspensions Over Time

**Figure 3:**
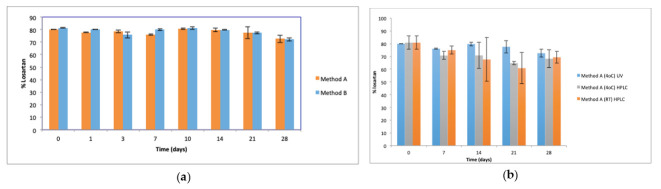
Losartan content of suspensions (*n* = 3, *±*SD, *p* > 0.05) (**a**) prepared by Methods A and B and stored at 4 °C, assayed by UV (**b**) prepared by method A, stored at 4 °C and RT, and analysed by UV and HPLC. Percent losartan content based on claimed losartan concentration.

The initial concentration of losartan in the oral suspensions, as determined by UV analysis, was 79.96 ± 0.05% and 81.4 ± 0.35% for methods A and B, respectively (t = 0). HPLC analysis of losartan content of suspensions prepared using method A was 80.8 ± 5.16%, confirming this reduced losartan content on day 0. UV analysis showed losartan content of suspensions prepared by methods A and B to be similar (Figure 3a) over the 28 days of storage and was in the range of 72.5–81.4% of the claimed losartan concentration (*p* > 0.05). HPLC analysis confirmed the losartan concentration of suspensions prepared by method A and stored at 4 °C over 28 days to be similar to the losartan content of suspensions stored at RT and to the losartan content of suspensions prepared by method A, stored at 4 °C and assayed by UV (Figure 3b).

### 2.3. Investigation of the Reduced Initial Losartan Concentration of Suspensions

#### 2.3.1. Analysis of Losartan Degradation

Eluates from HPLC analysis of losartan samples from the prepared suspensions at t = 0 were collected, lyophilised and analysed by ^1^H-NMR, and did not show any degradation products of losartan. Losartan reference standard subjected to forced degradation under oxidation conditions (3% *v/v* H_2_O_2_ for 7 days in the dark at RT) and analysed by HPLC analysis (Figure S1, Supplementary Materials) showed two small possible degradation peaks at retention times of 7.87 and 8.55 min. This represented a total degradation of 1.93% of losartan, assuming a similar response factor. For the losartan reference standard stressed in 1 M HCl for 14 days in the dark at 70 °C, two small potential degradation peaks at retention times of 9.80 and 12.54 min, representing a total degradation of 0.72% of losartan, were observed. The retention times of the degradation products did not interfere with the retention time for losartan standard at 6.09 min. HPLC eluates from the degradation samples, lyophilised and analysed by NMR did not identify any degradation products, although losartan in the samples was identified (Figure S2, Supplementary Materials).

#### 2.3.2. Effect of pH of Suspension Vehicle on Assayed Initial Losartan Content

Further investigation of the initial loss of losartan content was conducted by comparing the losartan concentrations of suspensions prepared with commonly used suspension vehicles; suspension diluent A, Ora-plus^®^:Ora-Sweet^®^ 1:1 with and without additional distilled water (method A and B vehicles, respectively), distilled water and suspension diluent A. UV assay of losartan content and pH of the suspensions prepared are shown in Table 1. Distilled water and suspension diluent A showed the greatest losartan content at 100 ± 7.74% and 93.19 ± 1.32%, respectively, on the day of preparation (day 0) compared with 79.96 ± 0.05 % and 81.38 ± 0.35 % (day 0) in 1:1 Ora-Plus^®^: Ora-Sweet^®^ preparations without and with distilled water, respectively. Interestingly, the pH of the suspensions prepared in distilled water and in suspension diluent A were significantly higher at 6.61 and 6.14, respectively, compared with a pH of 5.08 and 5.15 in method A and method B suspension vehicles. Losartan content of suspensions prepared with distilled water did not significantly decrease over 7 days storage at 4 °C and were 100 ± 7.74%, 92.16 ± 0.65% and 96.18 ± 0.36% at Days 0, 1 and 7, respectively.

### 2.4. Microbial Stability and Preservative Efficacy

Over the sampling period, the microbial testing confirmed that all suspensions were within the EP acceptable criteria for aqueous products for oral use. For all replicates, the TAMC observed after incubation for 1 and 5 days were between 0 and 10 colony forming units (CFU)/mL, well below the EP acceptance level of 1 × 10^2^ CFU/mL. For TYMC, the acceptance level of <10 CFU/mL was met and, notably, no colonies were observed on Sabaroud Dextrose Agar (SDA) plates from any formulation. The absence of *E. coli*, indicated by the absence of pink colonies on Brilliance UTI^TM^ agar, was also confirmed at all time-points. Losartan, stored unopened at 4 °C throughout the study period, was also within the EP acceptance criteria.

Losartan suspensions artificially contaminated with either *E. coli* or *S. aureus* produced bacterial counts of 63 ± 19 and 162 ± 47 CFU/mL, respectively, on day 1 (day of inoculation). These were reduced to 5 ± 4 and 32 ± 11, respectively, by day 2 and were absent by day 5 of testing.

## 3. Discussion

The objective of this study was to investigate the stability profile of extemporaneously prepared losartan potassium 5 mg/mL oral suspension, compounded by two methods A and B, over 28 days. To date, no published report of stability data is available for extemporaneously prepared losartan potassium 5 mg/mL oral suspension. All losartan potassium 5 mg/mL extemporaneous suspensions, prepared by both methods, were physically and microbiologically stable over the 28 days of storage at 4 °C and RT, and “closed” suspensions stored at 4 °C. The pH, colour and odour remained unchanged, the suspensions easily re-suspended on agitation and there was no visible degradation.

The content of losartan after preparation (day 0), prepared by both methods A and B, was found to be lower than expected at 80–81%, assayed by UV and confirmed by HPLC analysis. Subsequently, UV and HPLC analysis showed no significant difference in losartan content of suspensions stored at 4 °C and at RT over the 28 days storage. Chemical degradation of a drug may limit the shelf life of a preparation. A drug content ≥90% of the labelled amount is generally deemed to be acceptable [17]. No degradation of losartan was however detected by HPLC or NMR analysis of HPLC eluate of samples of losartan suspensions, in contrast to forced degradation products observed upon subjection of losartan potassium standard to either 3% *v/v* H_2_O_2_ or 1 M HCl at 70 °C, which resulted in degradation levels of 1.93% and 0.72%, respectively. Recently, a stability evaluation of losartan potassium solutions using a sensitive LC-MS/MS method has been reported [18], using as stressors 0.1 M HCl, 0.1 M NaOH, and 3% *v/v* H_2_O_2_. It was found that after 7 days at RT, degradation in the presence of 0.1 M HCl and 0.1 M NaOH was less than 1%, while in the presence of 3% *v/v* H_2_O_2_, degradation was about 10%. No difference in losartan content was also observed when suspensions were stored at the higher room temperature of ~25 °C. Forced degradation studies showed degradation of losartan, which were detected and measured by HPLC analysis. It is possible that the HPLC method used was not sensitive enough to detect losartan degradation of the suspensions examined. No reduction in losartan content of extemporaneous suspensions containing losartan potassium has been reported, and published monographs of losartan potassium 2.5 mg/mL extemporaneous suspensions assign a shelf-life of 28 days when stored under refrigerated conditions [10,11]. Preparation method B used in this study is similar to the methods reported in losartan potassium 2.5 mg/mL oral suspension monographs from the *US Pharmacist Journal* and The Hospital for Sick Children in Canada and The Nationwide’s Children’s Hospital Ohio, USA [10,11]. As per method B, all monographs include the addition of water to the losartan tablets before the addition of Ora-Plus^®^:Ora Sweet SF^®^. Method A preparation, used by Children’s Health Ireland at Crumlin, does not include the addition of water to the tablets/powdered tablets prior to adding the vehicle; however, UV analysis showed no significant difference in content over the 28 days storage was found between suspensions prepared by the two methods A and B.

The lower initial drug content was further investigated by preparing losartan potassium 5 mg/mL suspensions using distilled water and suspension diluent A as the suspension vehicles. The measured losartan contents at day 0 were higher at 100 *±* 7.74% and 93.19 *±* 1.32 %, respectively. This increase was related to the higher pH of 6.6 and 6.0 of the suspensions, respectively, compared with a pH of 5.08–5.15 for suspensions in Ora-Plus^®^ and Ora Sweet SF^®^ with and without distilled water, respectively. Losartan potassium has a pKa of 4.9 with a pH dependent solubility. In method A and method B, it is possible that losartan potassium from the 5 mg/mL suspensions is not completely extracted due to its lower solubility at acidic pH and/or possibly due to losartan precipitation occurring post extraction in the suspension vehicle. The pH of Ora-Plus^®^ and Ora-Sweet SF^®^ is acidic, in the range 4.0–4.5 and 4.0–4.4, respectively [19,20]. The international patent application WO 2009/112800 Al teaches losartan compositions suitable for oral administration where losartan is formulated in a suitable vehicle with a pH of below 7, to limit drug solubility and confer the benefit of taste masking of losartan, as well as to reduce potential of degradation of losartan over time [21].

In the development of Cozaar^®^ 2.5 mg/mL sachet for oral suspension, a low acidic pH was used for the suspension vehicle to limit the solubility of losartan potassium and hence taste mask the bitter taste of the drug [22]. The suspension vehicle used in Cozaar 2.5 mg/mL sachet for oral suspension is Ora-Blend^®^, which is a blend of Ora-Plus^®^ and Ora Sweet SF^®^ and has a pH of 4.2 [23]. In addition, at acidic pH, drug oxidation is reported to be reduced [19,20]. Interestingly, losartan content of the Cozaar^®^ sachet for oral suspensions assayed 5 min after preparation was >85% [22]. It is possible that due to the higher losartan concentration of 5 mg/mL of the suspensions studied, a higher proportion of the drug content is insoluble, resulting in the reduced losartan content of 80–81% observed after preparation. In suspensions prepared using distilled water at pH 6.5, >93% of losartan was assayed, and losartan content did not change over 7 days.

Reduced drug content can lead to inaccurate dosing and sub-therapeutic levels of the drug. Hypertension in children is most commonly caused by renal or vascular disease and tight management is necessary for initial treatment. Therefore, patients in this category would be particularly affected by inaccurate dosing [24]. As the dose in this case is titrated to response, the reduced initial content may not impact clinical response; however, dose variability may be an issue [25]. The inclusion of a wetting agent in the 5 mg/mL losartan potassium suspension may be necessary to aid drug dispersion and solubilisation in the vehicle or, alternatively, a vehicle with a higher pH could be examined.

Although the EP does not require an oral suspension to be free from microbes, resistance to microbial growth must be retained according to the specified criteria [26]. Losartan suspensions remained within the acceptable concentration of microorganisms (TAMC and TYMC) and *E. coli* was absent after storage for 28 days, irrespective of method of preparation and storage conditions. The inclusion of a suitable preservative is necessary to prevent microbial growth that may be introduced during use or may be present in the raw materials. The Ora-Plus^®^ and Ora-Sweet SF^®^ vehicles used in this work contain methylhydroxy- and propylhydroxy-benzoate preservatives. A 5-day study of preservative efficacy showed that following inoculation with marker organisms, the preparations were found to be free of both *E. coli* and *S. aureus* by day 5 of the study, confirming the efficacy of the preservatives.

## 4. Materials and Methods

### 4.1. Materials

Cozaar^®^ tablets 50 mg were obtained from Merck Sharp & Dohme Ltd., (MSD), Dublin, Ireland. Ora-Plus^®^ and Ora-Sweet SF^®^ were obtained from United Drug, Dublin, Ireland. Losartan potassium B.P. reference standard (≥99.5% purity) was obtained from Sigma-Aldrich, Arklow, Ireland. Suspension diluent A was purchased from Nova Labs, Wigston, UK. All materials used were HPLC or analytical grade. Potassium dihydrogen phosphate (Fluka Analytical, Loughborough, UK), sodium hydroxide, ortho-phosphoric acid, hydrochloric acid, hydrogen peroxide, acetonitrile and methanol (Lab-Scan analytical sciences, Dublin, Ireland) and distilled water (Fisher Scientific, Loughborough, UK) were used.

### 4.2. Extemporaneous Preparation of Losartan Potassium Oral Liquid Suspensions

Losartan potassium 5 mg/mL oral suspensions were extemporaneously compounded in 1:1 Ora-Plus^®^:Ora-Sweet SF^®^ using two preparation methods (Methods A and B). In method A, Cozaar^®^ tablets (5 × 50 mg) were ground to a fine uniform powder using a mortar and pestle before mixing with a small volume of 1:1 Ora-Plus^®^:Ora-Sweet SF^®^ and diluting it gradually with 1:1 Ora-Plus^®^:Ora-Sweet SF^®^ to a final volume of 50 mL. In Method B, Cozaar^®^ tablets were first dispersed in 5 mL distilled water, followed by the addition of premixed 1:1 Ora-Plus^®^ and Ora-Sweet SF^®^ to a final volume of 50 mL [10,11]. Suspensions were transferred to amber glass bottles and stored at 4 °C and at RT. Suspensions were prepared in triplicate. The suspensions were sampled and analysed on days 0, 1, 3, 7, 10, 14, 21 and 28. Suspensions were also stored unopened (closed) at 4 °C (“closed suspensions”), throughout the sampling period and analysed at day 28. All suspensions were shaken vigorously prior to sampling to ensure a homogenous aliquot was sampled.

### 4.3. pH, Visual Appearance, and Organoleptic Properties of Losartan Suspensions

The pH of each suspension was determined in triplicate immediately after preparation and on each sampling day using a calibrated pH meter (CyberScan 510, Lennox, Dublin, Ireland). At the same sampling points, the colour of the preparations was examined by visual inspection of a sample in a clear glass beaker against a black background. The odour, ease of re-suspension and presence/absence of sedimentation was also recorded. The suspension vehicle (1:1 Ora-Plus^®^:Ora-Sweet SF^®^) was used as the control.

### 4.4. Analysis of Chemical Stability of Losartan Suspensions

Chemical stability was investigated by ultraviolet (UV) spectrophotometry and by a stability indicating high performance liquid chromatography (HPLC) method as described previously [15,16]. The HPLC method was validated as per International Council fro Harmonisation of Technical Requirements for Pharmaceuticals for Human Use (ICH) guidelines [17]. For UV analysis, a 1 mL aliquot from each suspension was diluted in distilled water and its absorbance was read at 237 nm relative to losartan potassium reference standards. For HPLC analysis, the 1 mL aliquot was diluted with the mobile phase (potassium dihydrogen phosphate buffer (pH 6.0, 0.025 M) and acetonitrile (65:35, *v/v*). Chromatographic separations were performed on a Phenomenex C18 column (250 × 4.6 mm; 5 μm) at 40 °C using a HPLC system (Agilent HPLC 1120 Gradient, Agilent Technologies, Waldbronn, Germany) equipped with EZChrom Elite compact software version 3.3.2 and a UV detector. A flow rate of 1.5 mL/min was used for the mobile phase. Concentration of losartan in samples was estimated by peak area relative to the losartan potassium reference standard in the HPLC assay.

### 4.5. Forced Degradation Studies of Losartan Potassium

Losartan potassium reference standard (5 mg, *n* = 3) was stressed under two conditions in forced degradation studies; in 1 M HCl at 70 °C in the dark for 14 days (acid degradation) and in 3% H_2_O_2_ (*v/v*) at RT in the dark (oxidative degradation). Samples were assayed using HPLC. Aliquots of the eluate from HPLC for 7 days analysis, were collected, freeze-dried and subjected to NMR analysis to investigate the chemical structures/identities of the degradation products. Freeze dried samples were dissolved in 1 mL of deuterated DMSO (dimethyl sulfoxide-*d*_6_). NMR analysis was performed using a Bruker Avance 400 spectrometer (Bruker, Rheinstetten, Germany), recording ^1^H spectra at 400 MHz.

### 4.6. Analysis of Microbiological Stability of Losartan Suspensions

Microbiological assessment of suspensions was carried out on days 1, 8, 15, 22 and 29. Microbiological stability was confirmed if the EP acceptance criteria for microbiological quality of non-sterile aqueous products for oral use were met [26]. TAMC were determined by spreading duplicate aliquots (200 μL) from suspensions onto pre-poured Tryptic Soy Agar (TSA) plates (Serosep, Limerick, Ireland) and incubating at 37 °C for 5 days. TYMC were determined in duplicate by mixing 1 mL aliquots from suspensions with 35 mL volumes of warm liquid Sabaroud Dextrose Agar (SDA) (Oxoid, Basingstoke, UK). The warm mixtures were poured into Petri dishes, allowed to solidify at RT and incubated at 30 °C for 48 h. The absence of *E. coli* was determined in duplicate samples from suspensions by overnight enrichment of 200 μL aliquots in 5mL Tryptic Soy Broth (TSB) (Oxoid, Basingstoke, UK) at 37 °C and 150 rpm in a Gallenkamp Orbital Shaker Incubator (Fison Instrument Limited, Glasgow, UK). Aliquots (200 μL) from the enriched broths were spread onto Brilliance UTI™ agar (Oxoid, Basingstoke, UK) and incubated for 24 h at 37 °C. *E. coli* appears as magenta colonies on this agar. All plates were observed after appropriate incubation periods and colonies (if present) were counted and expressed as CFU/mL. “Spiked” samples were included for reference purpose to demonstrate CFU/mL above the EP acceptance criteria. *S. aureus* ATCC25923 for TAMC, *C. albicans* CA132A for TYMC and *E. coli* ATCC 25922 were added to vehicle samples to yield CFU/mL of >10^1^–10^2^, and these samples were tested for microbial stability along with test suspensions. Liquid and solid media were incubated without the addition of losartan/vehicle under the specified conditions as sterility controls.

### 4.7. Preservative Efficacy Testing

A 5-day study was carried out to determine the efficacy of the preservatives present in the preparation. The preservatives were methylhydroxybenzoate, propylhydroxybenzoate and potassium sorbate. Freshly prepared losartan suspensions (10 mL) were inoculated with approx. 100 CFU/mL *S. aureus* ATCC25923 (*n* = 3) and *E. coli* ATCC25922 (*n* = 3) (prepared by adding 100 μL of 1 × 10^5^-diluted bacterial suspensions of optical density (OD) 600 nm of 1.0). Duplicate 100 µL samples from each inoculated losartan suspensions were removed on days 0, 1, 2, 3, 4 and 5 and spread onto TSA plates and incubated at 37 °C for 24 h. CFU/mL were recorded. Non-inoculated losartan suspensions, sterility controls (TSA) and positive-growth controls were included.

### 4.8. Statistical Analysis

Statistical analysis was performed using GraphPad Prism (GraphPad, La Jolla, CA, USA) employing either *t*-test or ANOVA. *p* ≤ 0.05 was considered statistically significant.

## 5. Conclusions

The data from the present study support a 28-day physical and microbiological stability of losartan potassium 5 mg/mL extemporaneous oral suspensions prepared using Ora-Plus^®^ and Ora Sweet SF^®^. A reduced losartan content was measured at day 0 and this was related to the low solubility of losartan potassium in the acidic pH of Ora-Plus^®^ and Ora-Sweet SF^®^ vehicle used. No subsequent degradation or significant reduction in losartan content was measured in the suspensions, irrespective of the preparation method or storage conditions used. However, further investigations of the preparation method and losartan solubility in suspension vehicle are required to assign an evidence-based shelf-life to losartan potassium 5 mg/mL extemporaneous suspension, for increased confidence that the product is safe and effective over its shelf-life.

## Figures and Tables

**Table 1:** pH and losartan content of losartan suspensions prepared using various suspension vehicles at t = 0. Data presented are the mean of *n* = 3 ± SD.

Suspension Vehicle	pH	% Losartan Content
Distilled water	6.61 *±* 0.19	100.35 *±* 7.74
Suspension diluent A	6.14 *±* 0.10	93.19 *±* 1.32
1:1 Oraplus^®^:Orasweet^®^ (Method A Vehicle)	5.08 *±* 0.015	79.96 *±* 0.05
1:1 Oraplus^®^:Orasweet^®^ + DW^ 1^ (Method B Vehicle)	5.15 *±* 0.05	81.38 *±* 0.35

^1^ DW = deionised water.

## Data Availability

The data presented in this work are available on request from the corresponding author.

## Supplementary Materials

The following are available online: Figure S1. HPLC chromatogram of losartan potassium after stress testing (**A**) with 3% *v*/*v* H_2_O_2_ in the dark for 7 days at RT and (**B**) 1 M HCl in the dark at 70 °C for 7 days; Figure S2. ^1^H-NMR of recovered losartan.

## Sample Availability

Samples of compounds are not available from the authors.
