# Supplementary material for: Investigation of the Physical, Chemical and Microbiological Stability of Losartan Potassium 5 mg/mL Extemporaneous Oral Liquid Suspension
Source: Molecules. 2021 Jan 8;26(2):301. doi: 10.3390/molecules26020301 (PMC7827961; doi:10.3390/molecules26020301)
Supplement: Supplementary file 1 [file molecules-26-00301-s001.pdf]

Supplemental Files for:

Investigation of the Physical, Chemical and Microbiological Stability of Losartan Potassium 5 mg/mL Extemporaneous Oral Liquid Suspension

Lisa Foley [1,†], Jennifer Toney [1,†], James W. Barlow [2], Maura O'Connor [3], Deirdre Fitzgerald-Hughes [4] and Zebunnissa Ramtoola [1,*]

[1] School of Pharmacy and Biomolecular Sciences, RCSI University of Medicine and Health Sciences, 111 St. Stephen's Green, Dublin 2, Ireland; lisafoley@rcsi.ie; jennifertoney@alumnircsi.com

[2] Department of Chemistry, RCSI University of Medicine and Health Sciences, 123 St. Stephen's Green, Dublin 2, Ireland; jambarlow@rcsi.com

[3] Pharmacy Department, Children's Health Ireland (CHI) at Crumlin, Dublin 12, Ireland; maura.oconnor@olchc.ie

[4] Department of Clinical Microbiology, RCSI University of Medicine and Health Sciences, Education and Research Centre, Beaumont Hospital, Dublin 9. Ireland; dfitzgeraldhughes@rcsi.com

† These authors have contributed equally to this work.

* Correspondence: zramtoola@rcsi.com; Tel.: +353-1-402-8626

Method Validation

To establish linearity, the ICH guidelines recommend the use of at least 5 concentrations (0.1 mg/mL, 0.25 mg/mL, 0.5 mg/mL, 1.0 mg/mL, 2.0 mg/mL). Reproducibility of the calibration curve was evaluated by repeating the calibration using freshly prepared standard solutions on day 0, 7 and 14. The injection precision was evaluated by performing 5 replicate injections of the 1.0 mg/mL working standard solution to ensure repeatability.

Forced degradation studies

HPLC analysis of stressed losartan subjected to oxidation showed two small suspected degradation peaks (DP) with retention times of 7.867 and 8.547 min (as denoted by arrows in Figure. S1A). Following acid degradation of losartan, two small potential degradation peaks with retention times of 9.803 and 12.543 min were also observed. (Figure S1B).

A

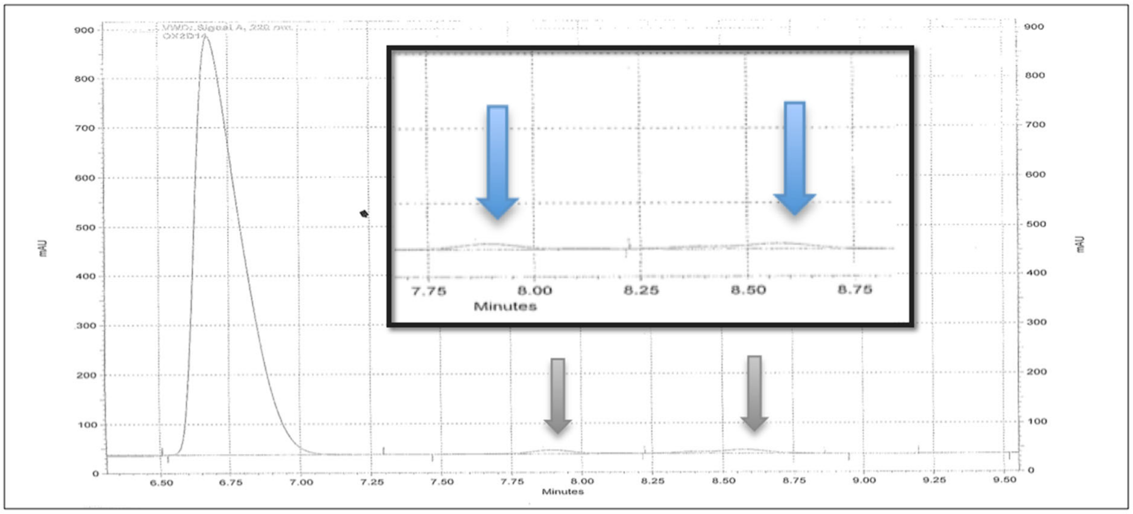

B

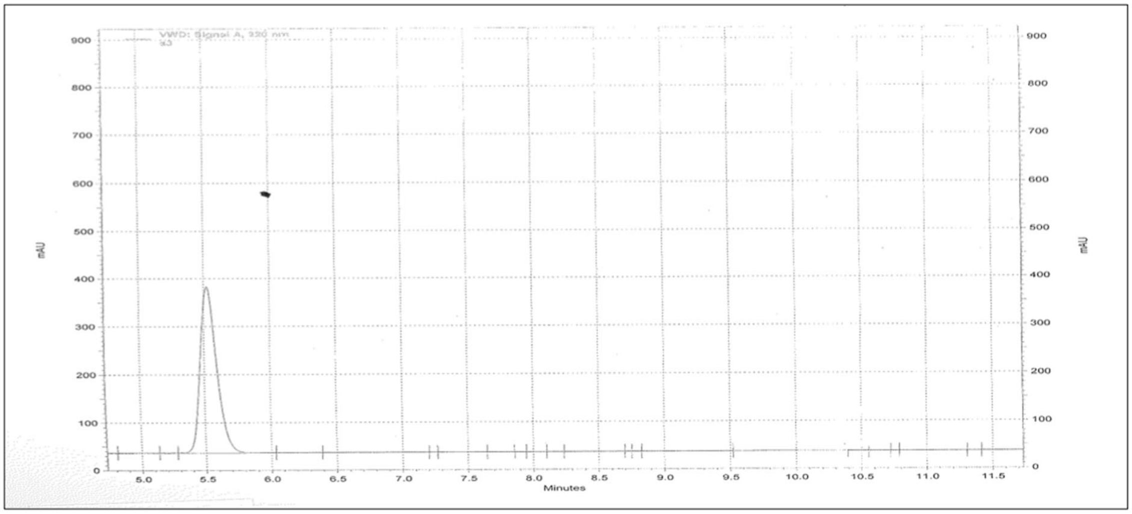

Figure S1. HPLC chromatogram of losartan potassium after stress testing (A) with 3% *v*/*v* H_2O_2 in the dark for 7 days at RT and (B) 1 M HCl in the dark at 70 °C for 7 days.

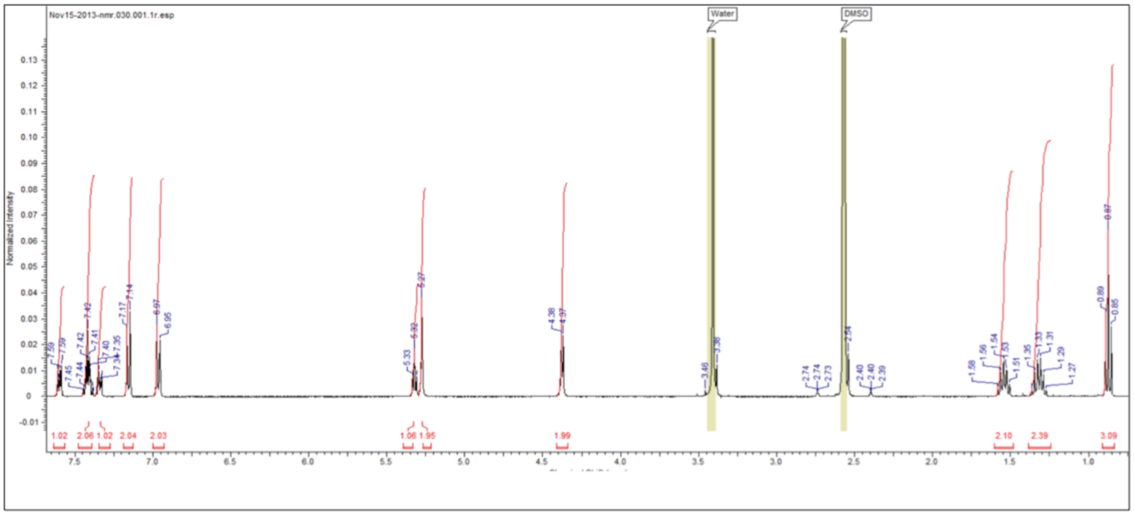

Figure S2. ^{1}H-NMR spectrum of recovered losartan.
